# Prediction of pyrotinib exposure based on physiologically-based pharmacokinetic model and endogenous biomarker

**DOI:** 10.3389/fphar.2022.972411

**Published:** 2022-09-23

**Authors:** Miao Zhang, Zhiheng Yu, Xueting Yao, Zihan Lei, Kaijing Zhao, Wenqian Wang, Xue Zhang, Xijing Chen, Dongyang Liu

**Affiliations:** ^1^ Drug Clinical Trial Center, Peking University Third Hospital, Beijing, China; ^2^ Institute of Medical Innovation, Peking University Third Hospital, Beijing, China; ^3^ School of Basic Medicine and Clinical Pharmacy, China Pharmaceutical University, Nanjing, China; ^4^ Department of Obstetrics and Gynecology, Peking University Third Hospital, Beijing, China; ^5^ Jiangsu Hengrui Pharmaceuticals Co, Ltd, Shanghai, China

**Keywords:** pyrotinib, PBPK model, clinical trial design, drug-drug interaction, 4βhydroxycholesterol, genotype

## Abstract

Pyrotinib, a novel irreversible epidermal growth factor receptor dual tyrosine kinase inhibitor, is mainly (about 90%) eliminated through cytochrome P450 (CYP) 3A mediated metabolism *in vivo*. Meanwhile, genotype is a key factor affecting pyrotinib clearance and 4β-hydroxycholesterol is an endogenous biomarker of CYP3A activity that can indirectly reflect the possible pyrotinib exposure. Thus, it is necessary to evaluate the clinical drug-drug interactions (DDI) between CYP3A perpetrators and pyrotinib, understand potential exposure in specific populations including liver impairment and geriatric populations, and explore the possible relationships among pyrotinib exposure, genotypes and endogenous biomarker. Physiologically-based pharmacokinetic (PBPK) model can be used to replace prospective DDI studies and evaluate external and internal factors that may influence system exposure. Herein, a basic PBPK model was firstly developed to evaluate the potential risk of pyrotinib coadministration with strong inhibitor and guide the clinical trial design. Subsequently, the mechanistic PBPK model was established and used to quantitatively estimate the potential DDI risk for other CYP3A modulators, understand the potential exposure of specific populations, including liver impairment and geriatric populations. Meanwhile, the possible relationships among pyrotinib exposure, genotypes and endogenous biomarker were explored. With the help of PBPK model, the DDI clinical trial of pyrotinib coadministration with strong inhibitor has been successfully completed, some DDI clinical trials may be waived based on the predicted results and clinical trials in specific populations can be reasonably designed. Moreover, the mutant genotypes of CYP3A4*18A and CYP3A5*3 were likely to have a limited influence on pyrotinib clearance, and the genotype-independent linear correlation coefficient between endogenous biomarker and system exposure was larger than 0.6. Therefore, based on the reliable predicted results and the linear correlations between pyrotinib exposure and endogenous biomarker, dosage adjustment of pyrotinib can be designed for clinical practice.

## Introduction

Pyrotinib is a novel irreversible epidermal growth factor receptor/human epidermal growth factor receptor 2 (EGFR/HER2) dual tyrosine kinase inhibitor, which can covalently bind to adenosine triphosphate binding sites of intracellular EGFR/HER2 to prevent self-phosphorylation or down-regulation of signal transduction, thereby inhibiting the growth of tumor. Currently, multiple clinical trials are evaluating the anticancer efficiency of pyrotinib in various solid tumors. Successfully, pyrotinib has obtained promising antitumor activity and acceptable tolerance in the treatment of patients with HER2-positive metastatic breast cancer and non-small-cell lung cancer harboring HER2 mutations (Ma et al., 2019; Zhou et al., 2020). With its unique advantages, pyrotinib has obtained the first global conditional approval for the treatment of HER2-positive, advanced or metastatic breast cancer in China (Blair, 2018; Ma et al., 2019).

Pyrotinib is a clinical oral dosage form with a therapeutic dose of 400 mg, and its bioavailability will be increased when taken with meals. The absorption of pyrotinib is slow with a median time to maximum plasma concentration (*T*_max_) of 3–5 h under different dosage regimens. Binding of pyrotinib to plasma proteins is strong (∼95%), leading to its wide distribution (Apparent volume of distribution, V_d_/F, ∼4000 L) *in vivo*. Mass balance study (CTR20170528) has shown that about 92.6% (Meng et al., 2019) of the radioactivity is recovered from excretions, including limited parent drug in feces (∼12.0% in fasted state and ∼3.27% in fed state) and urine (∼0.13%). Based on the biliary intubation experiment in rats, biliary excretion is likely to have minimal effects (∼0.16%) on pyrotinib clearance in human. Last but not least, metabolism plays the major role for pyrotinib clearance and cytochrome P450 3A (CYP3A), responsible for approximately 90% of pyrotinib metabolism, is the predominant isoform (Zhu et al., 2016).

Hence, pyrotinib may be a sensitive CYP3A substrate and drug-drug interaction (DDI) studies should be conducted in clinic according to the guidance of cytochrome P450 enzyme and transporter mediated drug interactions (Food and Drug Administration, 2020). Considering that physiologically based pharmacokinetic (PBPK) model can be used to narrow the knowledge gap between *in vitro* and *in vivo* (Grimstein et al., 2019), the pyrotinib PBPK model is proposed to develop firstly according to the preclinical data and limited clinical PK data, including single-ascending dose (SAD) and multiple-ascending dose (MAD) so as to support the clinical trial design of pyrotinib coadministration with perpetrators. Moreover, PBPK models can be used in lieu of some prospective DDI studies after the mechanistic model validated by pharmacokinetic (PK) data and information from DDI studies that used strong index perpetrators (Food and Drug Administration, 2020). Therefore, the DDI studies of pyrotinib *in vivo* are planned to conduct with strong inhibitor/inducer as the prospective clinical trials, and then use the PBPK model to estimate the effects of other CYP3A modulators on the exposure of pyrotinib *in vivo.* Likewise, patients with hepatic dysfunction or senile patients who have the weaken hepatic metabolic rate may have higher levels of pyrotinib exposure (Hunt et al., 1992; Elbekai et al., 2004), and evaluating the potential exposure of pyrotinib in these populations by the mechanistic PBPK model can provide scientific and reasonable suggestions for the designs of corresponding clinical trials.

Moreover, individual difference of CYP3A expression levels can also influence the exposure of substrate, which may be caused by changes in protein abundance under different genotypes (Tracy et al., 2016). CYP3A has several genetic variations and CYP3A4*1G, CYP3A4*18A and CYP3A5*3 have shown a high mutation frequency in the Chinese (Hu et al., 2005, 2006; Du et al., 2007), which can significantly impact the activity of isozymes (Kuehl et al., 2001; Fukushima-Uesaka et al., 2004). Cholesterol is catalyzed by CYP3A to form 4β-hydroxylcholesterol (4β-OHC) and the molar ratio (4β-OHC/CHO) of 4β-OHC to total cholesterol (CHO) is an endogenous biomarker of hepatic CYP3A activity (Bodin et al., 2001; Gravel et al., 2019). Thus, the relationships among pyrotinib exposure, genotypes and the biomarker are worthy of exploration to know the possible exposure in advance.

Herein, we are aimed to: 1) develop a basic pyrotinib PBPK model based on the limited clinical data to support clinical trial design of pyrotinib coadministration with strong perpetrator; 2) establish a mechanistic PBPK model to evaluate the potential exposure of pyrotinib under different DDI scenarios and in specific populations; 3) explore possible relationships among clearance of pyrotinib, gene polymorphism and biomarker, and 4) simulate pyrotinib exposure under various genotypes with corresponding protein abundance information *in vivo* to comprehensively integrate limitation and knowledge gaps and support reasonable dosage adjustment in clinical practice.

## Materials and methods

### Study strategy

**FIGURE 1:**
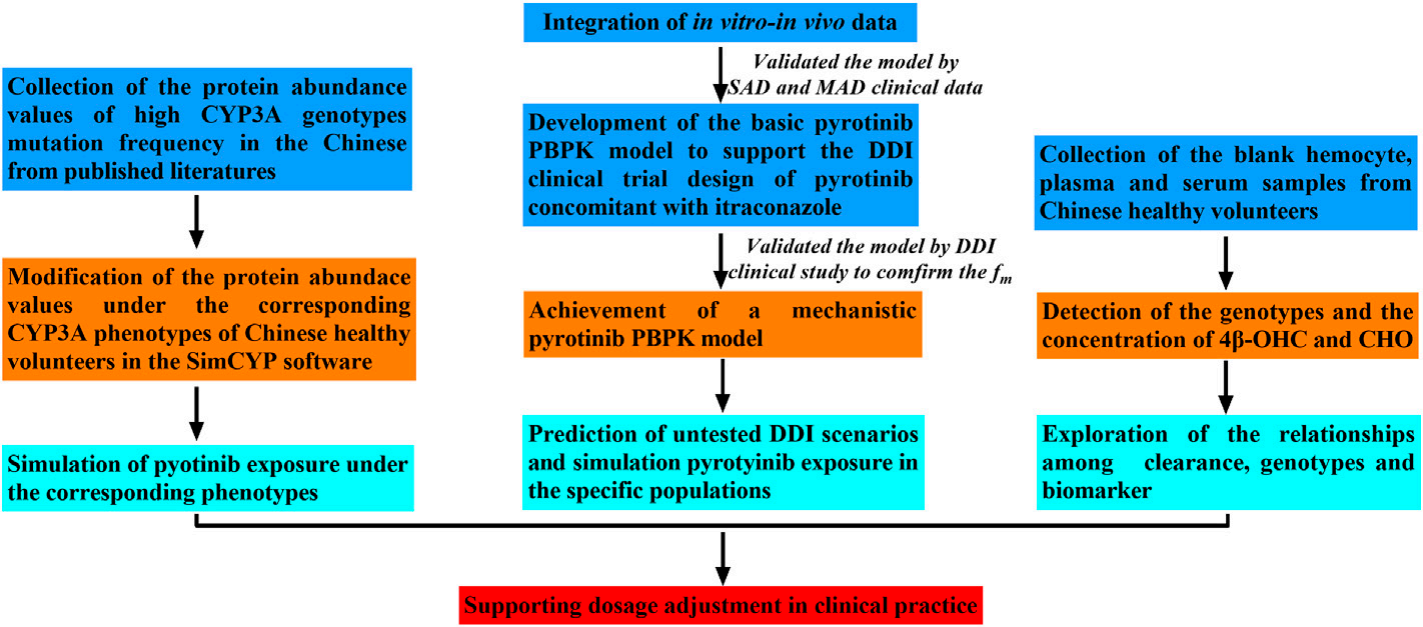
Strategy map for this study.

Firstly, the basic pyrotinib PBPK model was developed with the parameters both collected from preclinical and limited clinical data (SAD and MAD, NCT01937689) to describe the intracorporal process of the compound and then to support the DDI clinical trial design. Secondly, in order to improve the prediction performance of the model, and confirm the contribution of CYP3A to the metabolism of pyrotinib, the basic model was optimized and the mechanistic PBPK model was formed. Multi-dimensional validation was carried out on the mechanistic PBPK model, including not only SAD and MAD (NCT01937689) data, which were the same as the validation data of the basic model, but also the data of pyrotinib coadministration with capecitabine (NCT02361112) and pyrotinib coadministration with itraconazole (NCT04479891). Thirdly, untested scenarios including pyrotinib coadministration with CYP3A modulators, pyrotinib administration in hepatic dysfunction and elderly populations were simulated with the mechanistic pyrotinib PBPK model. Moreover, genotypes of CYP3A4*1G, CYP3A4*18A and CYP3A5*3, and the concentrations of 4β-OHC and CHO were also detected based on the blank hemocyte/plasma/serum samples from eighteen Chinese (NCT04479891). Meanwhile, based on the published protein abundance information of corresponding genotypes, the correlation between pyrotinib clearance and CYP3A5*3 allele was explored with the mechanistic model. Figure 1 displays the strategy diagram and Table 1 shows the simulation scenarios with the mechanistic PBPK model.

**TABLE 1:** Summary of the simulation scenarios in this study.

(A) Simulation scenarios of potential DDI			
Perpetrators	Dosage regimens	Treatment (days)	Pyrotinib dosage regimens
Itraconazole (Strong inhibitor)	200 mg (D1-D10, QD^a^)	10	400 mg on D4
Ketoconazole (Strong inhibitor)	200 mg (D1-D11, BID^b^)	11	400 mg on D5
Clarithromycin (Strong inhibitor)	250 mg (D1-D11, BID)	11	400 mg on D5
Erythromycin (Moderate inhibitor)	500 mg (D1-D11, QID^c^)	11	400 mg on D5
Diltiazem (Moderate inhibitor)	60 mg (D1-D11, TID^d^)	11	400 mg on D5
Fluconazole (Moderate inhibitor)	200 mg (D1-D14, QD)	14	400 mg on D8
Ciprofloxacin (Moderate inhibitor)	500 mg (D1-D11, BID)	11	400 mg on D5
Fluvoxamine (Mild inhibitor)	150 mg (D1-D11, BID)	11	400 mg on D5
Fluoxetine (Mild inhibitor)	40 mg (D1-D16, BID)	16	400 mg on D10
Efavirenz (Moderate inducer)	600 mg (D1-D15, QD)	15	400 mg on D9
(B) Simulation scenarios for specific populations with pyrotinib 400 mg			
Healthy Volunteers (20–50 years)			
Cirrhosis CP-A populations (20–50 years)			
Cirrhosis CP-B populations (20–50 years)			
Cirrhosis CP-C populations (20–50 years)			
Geriatrics populations (65–75, 75–85, and 85–95 years)			

a QD: quaque die.

b BID: bis in die.

c QID: qualer in die.

d TID: ter in die.

### Development of mechanistic pyrotinib PBPK model

Since the basic pyrotinib PBPK model was originally developed to support clinical trial design, and the mechanistic PBPK model has been validated with multiple clinical data, only the final model parameters are presented here. The mechanistic pyrotinib PBPK model was developed using advanced dissolution, absorption and metabolism (ADAM) model in the SimCYP Population-Based Simulator (Version 19, SimCYP Limited, Sheffield, United Kingdom, Certara Company). The permeability of compound in Caco-2 Transwell model and dissolution profile in aqueous buffer were used to develop the absorption model. Full-PBPK model was used to describe the distribution characteristics of pyrotinib. The volume of distribution at steady state (*V*_ss_) and partition coefficient of tissue to plasma (*K*_p_) were predicted. Meanwhile, *K*_p_ scalar of 1.80 was used to match the observed concentration-time profiles. Elimination of pyrotinib in PBPK model was jointly depicted by intrinsic clearance of human recombinant CYP isoforms (*CL*_int_), renal clearance (*CL*_R_), additional systemic clearance (*CL*_additional systemic_) and biliary clearance (*CL*_int_Bile_) in SimCYP. The contribution of each CYP isoform to the overall hepatic metabolism was calculated according to the protein abundance of the specific CYP450 isoforms in human liver and its corresponding *CL*_int_. Moreover, the contributions of kidney, unknown pathway and bile to the clearance of pyrotinib were obtained from the results of mass balance study in human (CTR20170528) and biliary intubation experiment in rats. Except for the *CL*_R_ (0.17 L/h) that were collected from clinical data, *CL*_int_Bile_ (0.4 μL/min/10^6^) and *CL*_additional systemic_ (4.5 L/h) were fitted according to the contribution percentage of pyrotinib clearance mentioned above and the PK profiles of pyrotinib in SAD clinical study. Physicochemical parameters, such as protein binding, B/P ratio and molecular weight (MW) were obtained from experimental results *in vitro*. Log*P* and *pK*_a_ were predicted based on the structure of pyrotinib. The detailed model parameters are summarized in Supplementary Table S1 of supplemental file.

### Model validation

The basic PBPK model was validated with limited clinical data, and the mechanistic PBPK model was fully validated with the clinical data that were obtained from the clinical studies of pyrotinib in healthy volunteers (SAD clinical study), the clinical studies of pyrotinib in breast cancer patients (MAD clinical study, NCT01937689), the efficacy study of pyrotinib coadministration with capecitabine (NCT02361112) and the DDI study of pyrotinib concomitant used with itraconazole (NCT04479891). The trial design in SimCYP Simulator was set to match population demographics (ethnicity, age and sex), dosage regimens and blood collection time points of each clinical study. The prediction performance of the mechanistic pyrotinib PBPK model was evaluated based on two criteria: 1) the observed values were within the 90% confidence interval (CI) of the predicted concentration-time profile; 2) the ratios of simulated area under the curve (*AUC*) and maximum concentration (*C*_max_) values to those observed values were within a predefined boundary of 0.5 to 2.0-fold. All simulations were performed based on 10 trials with 10 subjects (*n* = 100).

### Clinical trial design of pyrotinib coadministration with itraconazole

Considering the selectivity, safety, quantitative predictability and maximum inhibition of inhibitor (Ke et al., 2014), itraconazole was selected as the strong inhibitor in pyrotinib clinical DDI study. To identify the optimal design of itraconazole concomitant with pyrotinib in clinical DDI study, a series of dosage regimens of itraconazole and pyrotinib, including dose, interval and duration, were simulated with the basic PBPK model. Combined with the safety of pyrotinib and itraconazole, the predicted AUC ratio (AUCR) and *C*_max_ ratio (*C*_max_ R) of pyrotinib coadministration with itraconazole versus pyrotinib given alone were used as the reference for the design of dosage regimens. All simulations were performed based on representative population (SimCYP, Version 18). The simulated scenarios of itraconazole combined with pyrotinib are shown in Supplementary Table S2 of supplemental file.

### Pharmacokinetic DDI simulations

The mechanistic pyrotinib PBPK model was used to simulate the untested clinical DDI scenarios and estimate the effect of strong CYP3A inhibitors (clarithromycin, ketoconazole and itraconazole), moderate CY3A inhibitors (fluconazole, erythromycin, ciprofloxacin and diltiazem), mild CY3A inhibitors (fluvoxamine, fluoxetine and cyclosporine) and moderate CYP3A inducer (efavirenz) on the exposure of pyrotinib. “Chinese Healthy Volunteer” (20–50 years old, male:female = 1:1) and the default model of perpetrators within SimCYP were directly used for DDI simulations. To maximize the effect of perpetrators on pyrotinib, perpetrators were taken according to the clinical maximum dosage regimen, and a single dose of pyrotinib (400 mg, recommended dose in clinical treatment) was taken after the predicted plasma concentration of perpetrators reached the steady state. The detailed dosage regimens of untested DDI scenarios are shown in Table 1A.

### Simulation of pyrotinib administration in specific populations

The PK characteristics of pyrotinib in specific populations, including Chinese healthy volunteers, healthy volunteers (Caucasians), geriatrics and cirrhotic patients with Child-Pugh scores (CP) A, B and C (corresponding to mild, moderate and severe hepatic impairment) were simulated as well. As only 0.13% parent drug was excreted by kidney, the PK characteristic of renal impairment population was not estimated in this study. Except for Chinese healthy volunteers, other populations in SimCYP population library were Caucasians, so healthy volunteer (20–50 years old) was used as the baseline population. Geriatric population was divided into three subsets including 65–75, 75–85 and 85–95 age groups. For other specific populations, the simulated age range was set to 20–50 years old. Unless otherwise specified, physiological models within SimCYP database were directly used and all the simulations in this part included 10 trials with 10 subjects (*n* = 100) with the same proportion of male to female. Ultimately, the comparison of 400 mg pyrotinib system exposure in a specific population to that of baseline population (Healthy Volunteers of 20–50 years old, male: female = 1:1) was calculated in order to quantify the potential influence of internal factors on pyrotinib exposure. The detailed dosage regimens of simulations in specific populations are shown in Table 1B.

### Sample collection and detection of genotyping and endogenous biomarker

After overnight fasting, whole blood samples (before administration pyrotinib) were collected from 18 healthy volunteers participating in the DDI clinical study. The blood samples were immediately centrifuged at 2000×g, 4°C for 10 min. The hemocytes were collected for the detection of genotyping and the well-known single nucleotide polymorphisms (SNP) including CYP3A4*1G (rs2241480), CYP3A4*18A (rs28371759) and CYP3A5*3 (rs776746) were analyzed by polymerase chain reaction (PCR) and Sanger Sequencing. The content of serum CHO, one of the blood biochemical items, was detected during pre-enrollment screening (Day -2). The plasma samples were collected for the detection of 4β-OHC concentration by the method of LC-MS/MS. The detailed detection method of 4β-OHC concentration was as follows:

The ACQUITY UPLC^®^ I Class HPLC system (Waters, United States) connected to the API5500 Triple Quad mass spectrometer equipped with the Analyst 1.63 software (AB Sciex, Darmstadt, Germany) was used for the quantitative analysis. Chromatographic separation was performed on a XBridgeTM BEH C18 XP Column (4.6 × 75 mm, 2.5 μm, Waters). The mobile phases were (A) aqueous solution containing 0.1% formic acid (LC/MS grade, Fisher Scientific, United States) and (B) methanol (HPLC grade, Fisher Chemical, United States). Gradient elution was used as follows: 95% B at 0–4.9 min with a flow rate of 0.8 ml/min, 95%–60% B at 4.9–5.0 min with a flow rate of 0.8 ml/min, 60%–98% B at 5.0–5.3 min with a flow rate of 0.8 ml/min, 98% B at 5.3–5.9 min with a flow rate of 1.0 ml/min, 98%–95% B at 5.9–6.0min with a flow rate of 1.0 ml/min, 95% B at 6.0–8.0min with a flow rate of 0.8 ml/min. The injection volume was 5 μL. Mass spectrometric analysis was performed using positive ESI in multiple reaction-monitoring (MRM) mode. Ion transitions were m/z 385.3→109.1 and m/z 392.5→109.1 for 4β-OHC (Sigma-Aldrich/Avanti, Germany) and 4β-hydroxycholesterol-d7 (Internal standard, Sigma-Aldrich/Avanti, Germany), respectively. The analyte 4β-OHC displayed a good linearity at the range of 5–500 ng/ml. The developed method was validated according to the latest FDA guidelines for bioanalytical method validation.

### Prediction of pyrotinib exposure under various genotypes

Protein abundances of corresponding CYP3A4*1G, CYP3A4*18A and CYP3A5*3 genotypes in Chinese liver were retrieved from PubMed database with the keywords of “CYP3A4*1G/CYP3A4*18A/CYP3A5*3”, “protein abundance” and “Chinese volunteers”. Considering the inter-individual variation, protein abundance of individuals was obtained from literature (Xiao et al., 2019) by Plot Digitizer (Version 2.26, GetData, China). Variable coefficients were also calculated based on the observed values. The trial design in SimCYP Simulator was set to match the clinical study and the simulations were conducted based on 10 trials with 10 subjects (*n* = 100). In addition to changing the abundance of corresponding isoenzyme in the liver of Chinese physiological model, other parameters were directly used.

### Statistical analyses

The statistical analyses about significance levels and correlation analyses were assessed with the GraphPad Prism Version 8.0 (GraphPad Software, San Diego, CA, United States), and *p*-value < 0.05 was used to estimate the significance.

## Results

### The dosage regimen of pyrotinib coadministration with itraconazole

Various dosage regimens (See Supplementary Table S2) of pyrotinib concomitant with strong inhibitor, itraconazole, were simulated with the basic PBPK model to explore the “worst-case” DDI scenario. The predicted DDI magnitude increased with the duration of itraconazole treatment, and the loading dose of itraconazole on the first day could also increase the inhibition of CYP3A4. Meanwhile, the increase of pyrotinib dose could not alter the value of *AUC*R. Since the predicted *C*_max_R was lower than two and the clinical dose of pyrotinib was 400 mg per day, the low-dose pyrotinib (80 mg, single dose) and itraconazole (200 mg, QD for 17 days), dosage regimen G in Supplementary Table S2 of supplemental file, were used in the DDI clinical trial to obtain the potential maximum DDI effect while ensuring the safety and tolerability of subjects (Liu et al., 2021).

### PBPK model development and validation

The mechanistic pyrotinib PBPK model was developed and validated based on preclinical and clinical data. The final pyrotinib PBPK model parameters were presented in Supplementary Table S1. The observed and simulated geomean plasma concentration-time profiles for single or multiple dose(s) of pyrotinib were shown in Supplementary Figures S1–S3. Ratios (predicted value vs*.* observed value) of main pharmacokinetic parameters were approximate to 0.80–1.25 folds (See Supplementary Figure S4). All of them indicated that the model could well capture the absorption, distribution and elimination characteristics of pyrotinib *in vivo*. In addition, the mechanistic PBPK model was also validated by the data from clinical DDI study. The predicted and observed pyrotinib concentration-time profiles in the presence and absence of itraconazole were well matched and presented in Supplementary Figure S5, and the ratios of PK parameter between predicted values and observed values were within 0.80–1.25 folds. Meanwhile, the predicted metabolism fraction (*f*_m_) of CYP3A4 to the total clearance of pyrotinib was consistent with the experimental results (Zhu et al., 2016), which illustrated that pyrotinib PBPK model was a mechanistic model and could be used to simulate preset scenarios without further modification. The predicted contribution percentage of each organ/CYP isoform was shown in Supplementary Figure S6.

### Evaluating the effects of perpetrators on pyrotinib exposure

**FIGURE 2:**
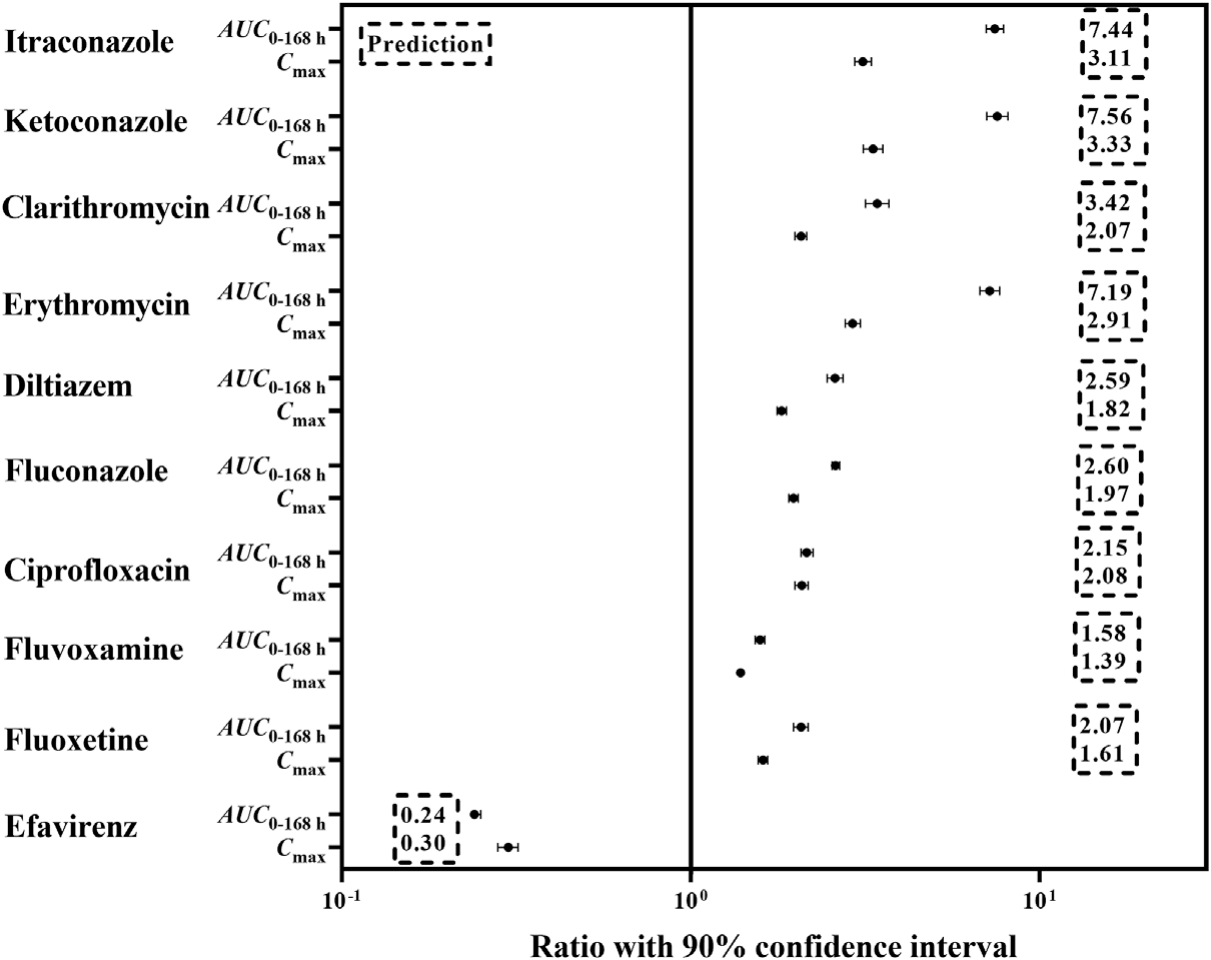
The predicted change in exposure after pyrotinib coadministration with potential CYP 3A perpetrators.

The mechanistic PBPK model was used to evaluate the effects of perpetrators (CYP3A inhibitors and inducers) on pyrotinib exposure in “Chinese healthy volunteer” aged 20–50 years with the same proportion of male and female. In the presence of strong CYP3A inhibitors itraconazole, ketoconazole and clarithromycin, the *AUC*_0–168 h_ of pyrotinib (400 mg, clinical dosage) was increased by 7.4-fold, 7.5-fold and 3.4-fold, respectively, compared with that of 400 mg pyrotinib alone. Although clinical DDI study indicated that itraconazole could increase the systemic exposure of pyrotinib (80 mg) by 11.8-fold, this was obtained by comparing the *AUC*_0–168 h_ of pyrotinib in the DDI clinical study with the *AUC*_0–24 h_ of pyrotinib (80 mg) administrated alone. Actually, under the same comparison criteria (*AUC*_0–24 h_), pyrotinib exposure could be increased by 6.36-fold in the presence of itraconazole. Therefore, there was no contradiction between the predicted and observed values of pyrotinib exposure in the presence of itraconazole. The pyrotinib exposure increased by 7.1-fold, 2.5-fold, 2.6-fold and 2.1-fold when combined with erythromycin, diltiazem, fluconazole and ciprofloxacin, respectively. However, erythromycin was an exception as its effect on pyrotinib exposure was similar to itraconazole. In fact, erythromycin was a time-dependent CYP 3A inhibitor, which had a potent impact on CYP3A isoenzyme (Yadav et al., 2018). The mild CYP3A inhibitors might also produce significant effects on the pyrotinib disposition due to that the predicted systemic exposure increased by 1.58-fold and 2.07-fold when pyrotinib combined with fluvoxamine and fluoxetine, respectively. CYP3A moderate inducer, efavirenz, appeared to significantly reduce the pyrotinib exposure (76%). Detailed results were shown in Figure 2.

### Simulation results of pyrotinib exposure in specific populations

**FIGURE 3:**
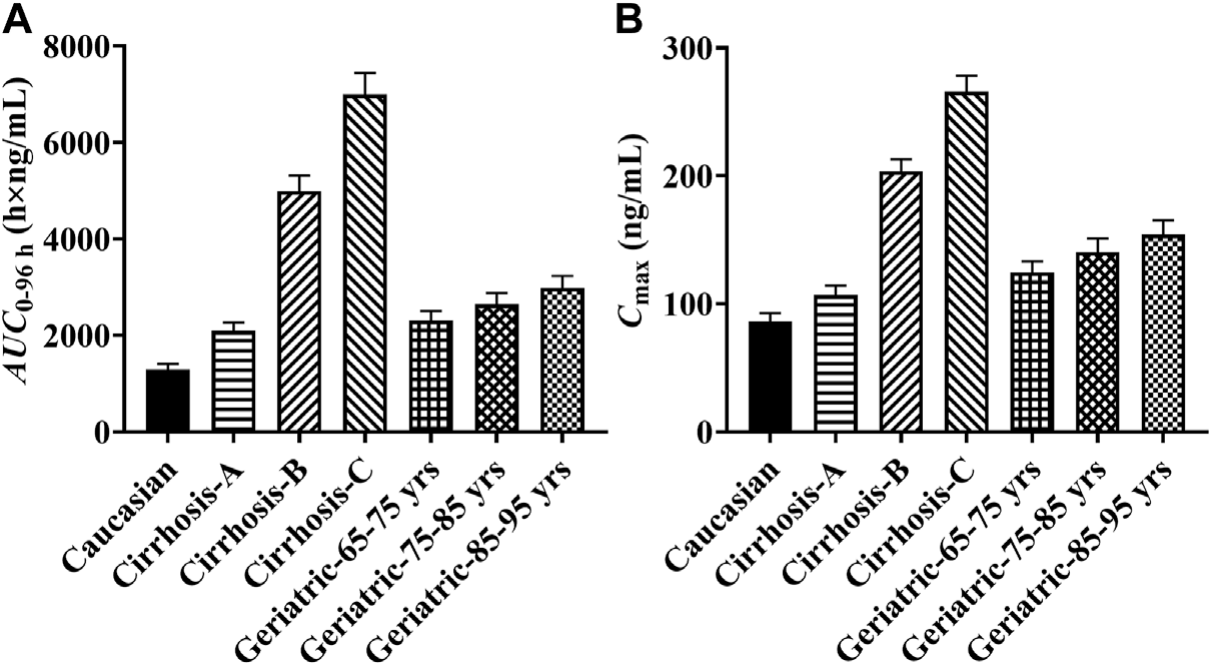
The predicted pyrotinib exposure in specific populations. (Note: yrs: years old; **(A)** comparison of pyrotinib *AUC*_0-96h_ in specific populations; **(B)** comparison of pyrotinib *C*_max_ in specific populations).

Since pyrotinib was mainly metabolized by the liver, the exposure of pyrotinib in the liver impairment and geriatrics was estimated. Compared with the baseline population, the liver impairment patients with CP score A, B and C had the increasing exposure along with the progression of disease, and the ratios of the main PK parameters (*AUC*_*0-96*_ **_*h*_ and *C*_max_) ranged from 1.62 to 5.42 and 1.24 to 3.09, respectively. The exposure of pyrotinib in geriatric population (aged 65–95 years) increased with age, and the detailed *AUC*_*0-96*_ **_*h*_ increments were 79, 105 and 131% corresponding to geriatric with 65–75, 75–85 and 85–95 years old, respectively. Results were exhibited in Figure 3.

### Relationships amont exposure, genotypes and endogenous biomarker

**FIGURE 4:**
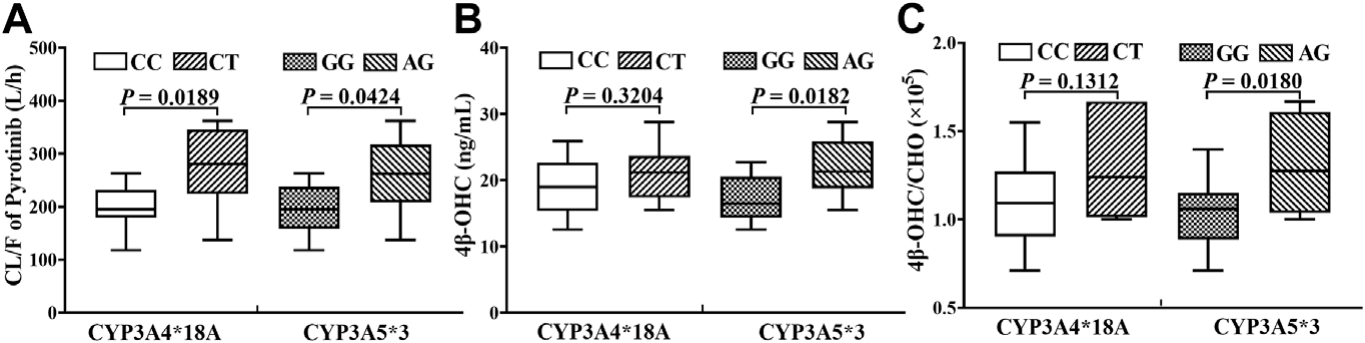
The statistical analyses results about the influence of genotypes on pyrotinib clearance **(A)** and endogenous biomarker **(B, C)**.

**FIGURE 5:**
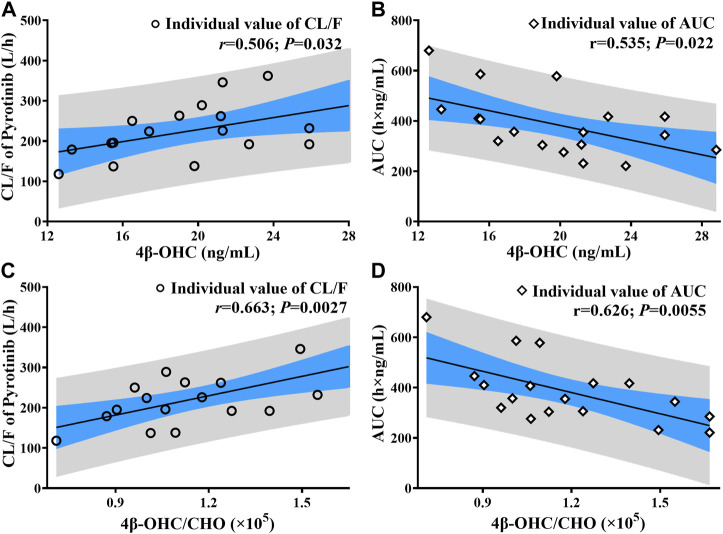
The correlation analysis results between pyrotinib clearance/exposure and endogenous biomarker (4β-OHC and 4β-OHC/CHO) (The black line: Linear fitting for individual value; r: correlation coefficient; P: statistical analysis results; **(A, C)** correlation analysis results between pyrotinib clearance and endogenous biomarker; **(B, D)**: correlation analysis results between pyrotinib AUC and endogenous biomarker).

In the limited samples, CYP3A4*18A (rs28371759) and CYP3A5*3 (rs776746) exhibited high mutation frequency, while CYP3A4*1G (rs2241480) showed a monomorphic genotype. The clearance of pyrotinib in individuals with different genotypes was statistically significant (Figure 4A), and only the CYP3A5*3 gene mutation had the notable influence on 4β-OHC concentration or the molar ratio of 4β-OHC to CHO (Figures 4B,C). Moreover, a genotype-independent linear correlation was found between exposure/clearance of pyrotinib and 4β-OHC concentration, and the correlation coefficient (*r*) was about 0.535, *p* < 0.05 (Figure 5A). Actually, there was a better genotype-independent linear correlation between the exposure/clearance of pyrotinib and 4β-OHC/CHO (*r >* 0.6, *p* < 0.05; Figure 5B). Therefore, the CYP3A basal expression level indeed had an impact on system exposure and plasma 4β-OHC concentration, and the ratio of 4β-OHC to CHO could be used as an indicator for pyrotinib exposure.

### Exploring the effect of genotypes on the clearance of pyrotinib with the PBPK model

**FIGURE 6:**
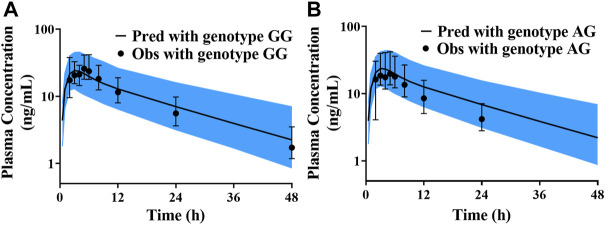
The validated results for pyrotinib exposure under different CYP3A5^*^3 genotypes **(A)** simulation and validated with the genotype of CYP3A5^*^3/^*^3; **(B)** simulation and validated with the genotype of CYP3A5^*^1/^*^3).

There were few published data about the protein abundance of CYP3A4*18A in the Chinese, and the median protein abundance values of corresponding CYP3A5*1/*1, CYP3A5*1/*3 and CYP3A5*3/*3 in Chinese population were obtained from reference (Xiao et al., 2019). The collected data were separately put into the liver module of Chinese population database to predict the *CL*/F of pyrotinib at different genotypes. Due to the small sample size (n = 18), only CYP3A5*1/*3 and CYP3A5*3/*3 genotypes were detected among those hemocyte samples. Thus, the predicted concentration-time profiles were validated according to the limited data. All of the observations were within the 90% confidence interval of the predicted values, which exhibited good prediction performance under corresponding protein abundance values. However, there was no significant difference in *CL*/F among different genotypes, and the *p-*value was 0.85. Validation results were shown in Figure 6.

## Discussion

Although the basic PBPK model was not validated by PK data from the DDI study, the results of mass balance study and the renal excretion fraction in human are known (Meng et al., 2019), which provide the clues for speculating the fraction of metabolism. Moreover, combined with the contribution of CY3A to the metabolism (90%) (Zhu et al., 2016) and the intrinsic clearance of CYP3A to pyrotinib metabolism *in vitro*, the metabolism and elimination characteristics of pyrotinib were primary understood. To simplify the basic PBPK model and ensure the accuracy of metabolism and elimination evaluation, the first-order absorption model was used to describe the absorption characteristics by fitting the absorption parameters. After the basic PBPK model could capture the PK profiles of pyrotinib in SAD and MAD clinical trials with the predicted *f*_m_ of approximately 96.5%, the model was used to simulate various DDI dosage regimens. Actually, the predicted AUCR (population representative) was slightly overestimated observed value, and then the mechanistic PBPK model was established based on multiple *in vitro* and *in vivo* data.

With the “bottom-up” method, the mechanistic pyrotinib PBPK model was developed, which was able to capture the absorption and disposition characteristics of pyrotinib in healthy subjects and HER2 positive breast cancer patients at single dose, multiple-dose and DDI scenarios. Thus, the PBPK model can be used to quantitatively evaluate the influences of CYP3A perpetrators on pyrotinib exposure. As pyrotinib is mainly metabolized by CYP3A isoenzymes (*f*_m_, ∼90%) *in vitro* (Zhu et al., 2016), the potential inhibition may be exhibited after pyrotinib coadministration with erythromycin (CYP3A moderate inhibitor). Coincidentally, after coadministration with erythromycin (500 mg, three times a day) (Olkkola et al., 1993), the exposure of CYP3A substrate midazolam (*f*_m_, ∼96%) increased by 4.42-fold, which was similar to the 3.40-fold increase of pyrotinib exposure at the same dosing interval. Although clarithromycin is a strong CYP3A inhibitor, the 3.42-fold increment of pyrotinib exposure is less than that of itraconazole. Actually, clarithromycin (250 mg, twice daily) could enhance midazolam exposure by 3.57-fold (Yeates et al., 1996), and the megadose clarithromycin (2,500 mg, twice daily) could improve midazolam exposure by a factor of 7 (Gorski et al., 1998). Therefore, the DDI predicted results in this study are reliable for guiding dose adjustment. Though, the pyrotinib PBPK model was not validated by the clinical data of CYP3A inducer, the predicted low exposure of pyrotinib after coadministration with efavirenz also suggested that the potential impact of moderate CYP3A inducer on the pyrotinib efficacy should not be ignored, and the predicted result has been used as a reference for the design of corresponding clinical trial (NCT04680091).

In fact, the effect of rifampin, a strong CYP3A inducer, on the exposure of pyrotinib was also estimated *in vivo,* and the *AUC* and *C*_max_ of pyrotinib were reduced by 96 and 89%, respectively. However, pyrotinib PBPK model is inadequate to predict the effect of rifampin on pyrotinib exposure with the default rifampin model in SimCYP. Considering that the lower exposure may lead to inefficacious concentration and the *f*_m_ of pyrotinib PBPK model has been verified by the clinical data of itraconazole concomitant used with pyrotinib, the rifampin model is no longer optimized. Actually, the rifampin model in the SimCYP software cannot accurately capture the observed concentration-time profiles in some case (Wagner et al., 2016) because of the complex drug interaction mechanisms associated with rifampin, such as the induction of CYP3A isoform and intestinal P-gp in human by rifampin (Greiner et al., 1999; Westphal et al., 2000).

The mass balance (400 mg, single dose) study in human subjects showed that after administration pyrotinib under fed state, about 0.13 and 3.27% parent drug were secreted and excreted through urine and feces, respectively. In addition, about 0.16% of the parent drug was excreted by bile in rats. Therefore, P-gp has little effect on the elimination of pyrotinib in the kidney and liver. Although pyrotinib is a compound with weak permeability, food can significantly reduce the excretion of pyrotinib compared with taking pyrotinib under fasted state (∼12%). Hence, intestinal P-gp has the finite role in the absorption of pyrotinib under fed state. As rifampin was concomitant administrated with pyrotinib under fed state, the low exposure of pyrotinib might be caused by the overexpression of CYP3A. Although itraconazole can also inhibit the efflux of P-gp (Mathis and Friedman, 2001), the limited inhibition may be caused by itraconazole according to the above discussion.

Sensitive analyses of the unmeasured parameters, such as log *P*, *CL*_additional systemic_ and *CL*_int_Bile_ were performed to estimate their rationality. Meanwhile, the importance of P-gp transporter was also assessed. Noteworthily, all of these parameters had no significant effects on the prediction performance of pyrotinib exposure. Considering the robustness of pyrotinib PBPK model and the pyrotinib disposition characteristic, the pyrotinib PK in liver impairment and geriatric populations were simulated. Because the specific populations in SimCYP population library were developed based on Caucasians, pyrotinib exposure under 400 mg (recommended dosage in clinical practice) in healthy subjects (20–50 years old) was served as a reference for safety. Since genotype-independent linear correlation exists between dose and exposure, dosage adjustment can be designed according to the predicted results for future clinical trials in liver impairment and geriatric populations.

Genotypes of CY3A4*18A (rs28371759) and CYP3A5*3 (rs776746) have shown high-frequency in the Chinese (Hu et al., 2005), and can impact the clearance of pyrotinib according to the results of a clinical trial in eighteen Chinese healthy volunteers after taking 80 mg of pyrotinib. The gene mutation of CY3A4*18A has the subtle influence on 4β-OHC content, and whether the molar ratio (4β-OHC/CHO) can be used as the biomarker of CYP3A4*18 metabolic activity may depend on the type of substrate (Kang et al., 2009). Although the samples used for the detection of 4β-OHC and CHO contents were not collected at the same time, both samples were taken from the same person who fasted overnight. Moreover, 4β-OHC has a long half-life (about 60 h) (Bodin et al., 2002) leading to a stable content of 4β-OHC *in vivo.* Therefore, with the genotype-independent linear correlation between endogenous biomarker and pyrotinib exposure/clearance, the possible system exposure can be obtained in advance to provide a reference for precision medication.

Based on the limited protein abundance values of CYP3A5*3 mutant and wild types in Chinese population, the pyrotinib exposure was simulated (10 trials with 10 subjects) under the corresponding CYP3A5 protein abundance values. However, the predicted results were almost the same among the genotypes of CYP3A5*1/*1, CYP3A5*1/*3 and CYP3A5*3/*3. The phenotype of CYP3A5 isoenzyme is related to the expression of CYP3A5*3 allele, which can encode an abnormal splicing mRNA with a premature termination codon, resulting in the loss of CYP3A5 expression (Kuehl et al., 2001) and then reduce the clearance of substrate compound. However, there exists a correlation between the abundance of CYP3A4 and CYP3A5 (Lin et al., 2002), and the pyrotinib is rarely metabolized by CYP3A5 isoform, which may lead to the approximate simulation results. Moreover, both CYP3A4*18A and CYP3A5*3 genotypes are mutants, and the heterozygotes of CYP3A4*18 A (7/18) and CYP3A5*3 (9/18) are found in the same individual, which may result in the high clearance with the large abundance value of CYP3A4 and CYP3A5 *in vivo*. Nevertheless, the observed geomean *AUC* ratio of homozygote to heterozygote is 1.31, indicating that the mutant genotypes cannot produce powerful effects on the safety. Hence, there is no need to pay too much attention to the genotypes of CYP3A.

Throughout the approved new drugs by FDA between 2015 and 2019, mechanistic PBPK model has become an effective means to help applicant to waive some DDI clinical trials. Actually, in the early stages of DDI clinical trial design, the pyrotinib PBPK model, validated only by SAD and MAD clinical data, was used to simulate potential DDI scenarios to help researchers develop a reliable dosage regimen. Hence, PBPK model is a useful tool in the whole process of new drug development. Likewise, in this study, the mechanistic pyrotinib PBPK model would allow a conservative evaluation for the effects of CYP3A inhibitors on pyrotinib exposure. Meanwhile, the predicted pyrotinib exposure in the specific populations including liver impairment and geriatric can also be used as a reference for clinical trial in the future. Furthermore, the genotype-independent linear correlation between endogenous biomarker and pyrotinib exposure can provide a simple and convenient method to predict the system exposure in advance. Due to the underestimation, the model is inadequate to predict the effects of CYP3A inducer on the pyrotinib exposure. Actually, rifampin, a strong CYP3A inducer, has the unlimited potential to induce the expression of CYP isoforms and transporters, which exceed the predictive power of rifampin model built-in SimCYP, such as abemaciclib (Posada et al., 2020), palbociclib (Yu et al., 2017), ixazomib (Gupta et al., 2018), upadacitinib (Food and Drug Administration, 2019) and so on. Therefore, it is crucial and urgent to develop a verifiable rifampin model with multiple inductive mechanisms. In addition, PBPK model is sufficient to simulate the effects of gene mutations on the exposure of one compound (Food and Drug Administration, 2018). However, limited protein abundance values and small sample sizes genetic testing may result in insignificant differences in the predicted results. Thus, it is necessary and meaningful to study the correlation between CYP3A4*18A genotype and protein abundance in the future.

## Conclusion

Pyrotinib PBPK model is used to support clinical trial design and new drug marketing application, providing a successful case for the application of PBPK model in new drug development. Although, the basic pyrotinib PBPK model was developed without the validation of DDI clinical data, the mass balance study *in vivo* and metabolic study *in vitro* were the fundamental data to ensure the accuracy of the disposition. Subsequently, the mechanistic PBPK model was established, and validated by the clinical DDI PK data. Therefore, the mechanistic model can be used to explore untested scenarios such as DDI and taken in specific populations to assist in dosage regimen design, inform regulatory decisions and support drug labeling. Moreover, the genotype-independent linear correlation coefficients between biomarker and the system exposure of pyrotinib were obtained, which provided another approach for scientific exploration of pyrotinib exposure. Therefore, dosage adjustment of pyrotinib can be designed by the mechanistic PBPK model and endogenous biomarker in clinical practice.

## Data Availability

The original contributions presented in the study are included in the article/Supplementary Material, further inquiries can be directed to the corresponding author.
