# Supplementary material for: Prediction of pyrotinib exposure based on physiologically-based pharmacokinetic model and endogenous biomarker
Source: Front Pharmacol. 2022 Sep 23;13:972411. doi: 10.3389/fphar.2022.972411 (PMC9543720; doi:10.3389/fphar.2022.972411)
Supplement: Supplementary file 1 [file DataSheet1.pdf]

Figure

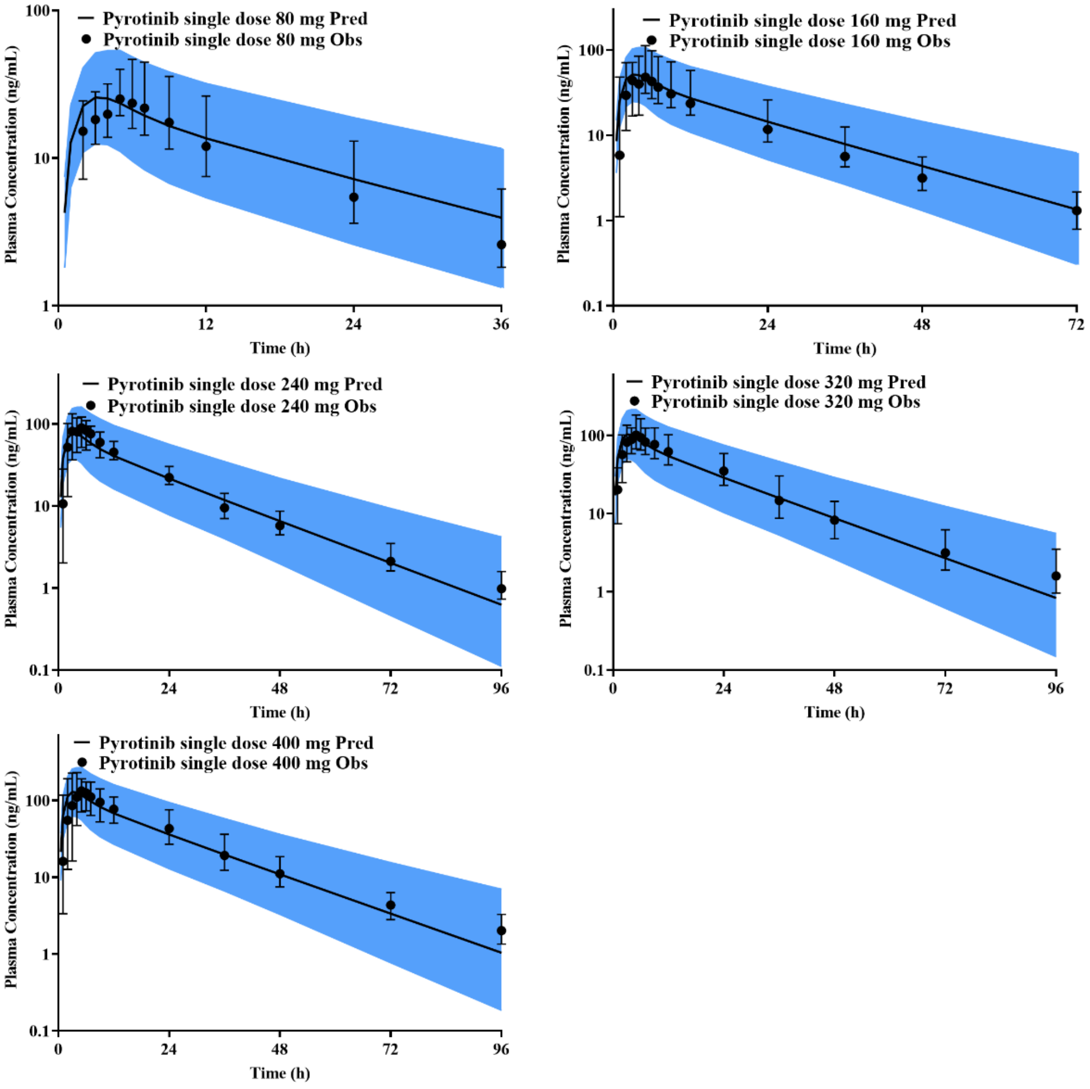

Figure S1 Validated results for pyrotinib SAD study (The black line is the predicted value, the dark spots are the observed values, and the blue range is 90% CI of the predicted results)

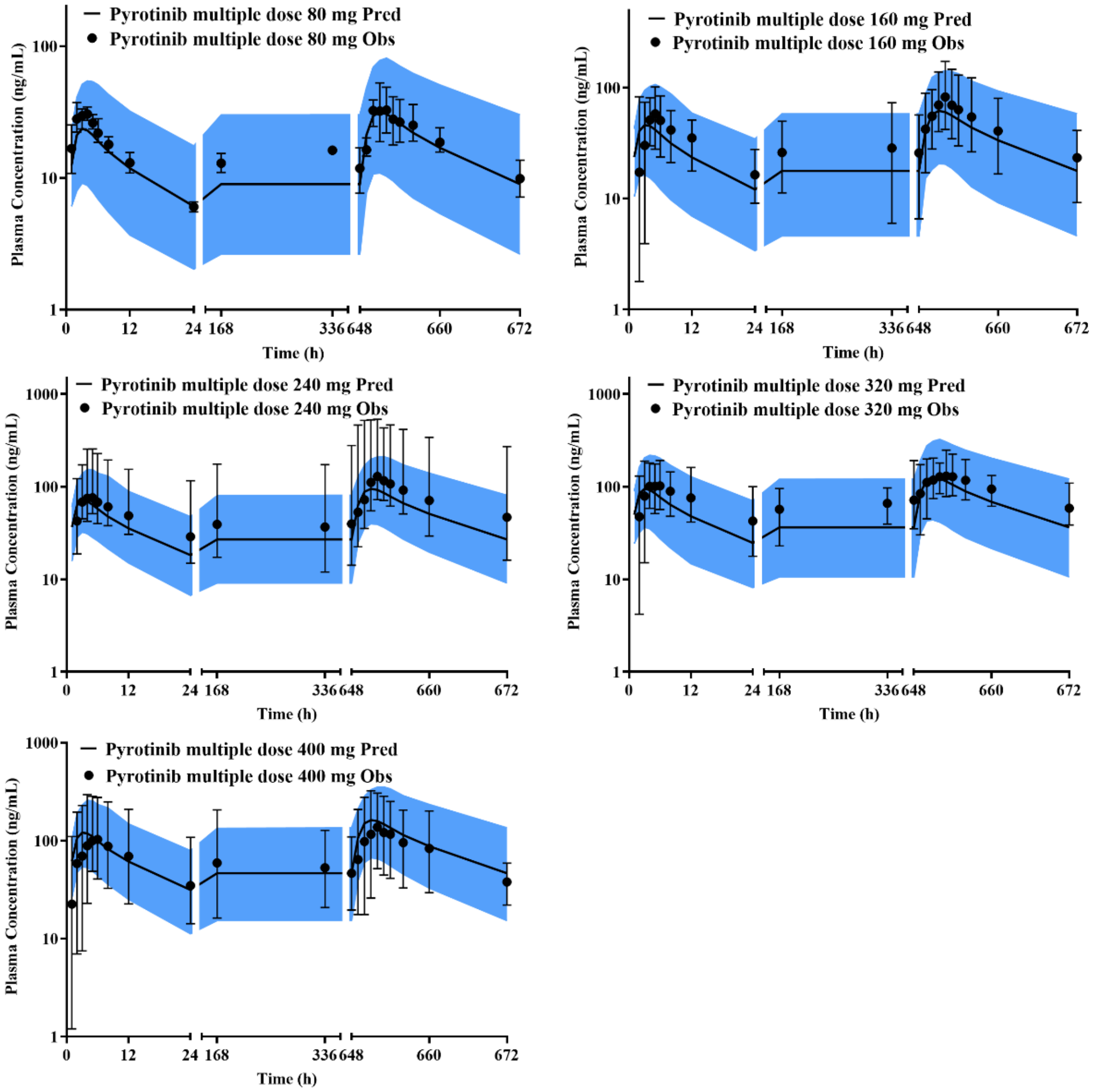

Figure S2 Validated results for pyrotinib MAD study (The black line is the predicted value, the dark spots are the observed values, and the blue range is 90% CI of the predicted results)

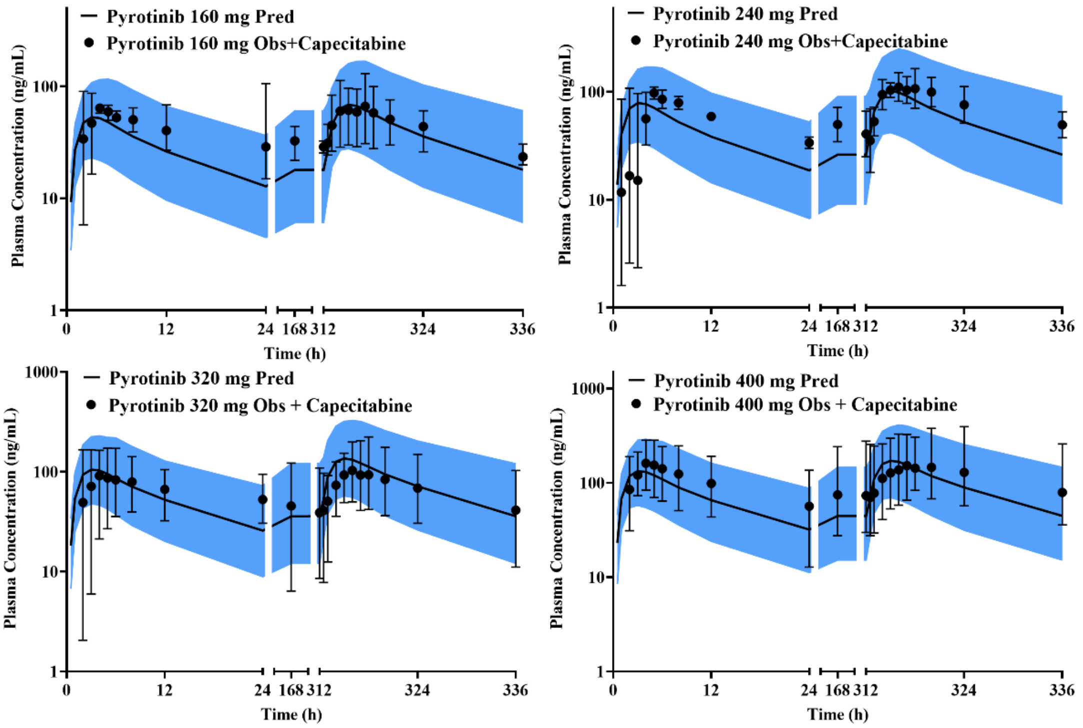

Figure S3 Pharmacokinetic validated results for pyrotinib coadministration with capecitabine (The black line is the predicted value, the dark spots are the observed values, and the blue range is 90% CI of the predicted results)

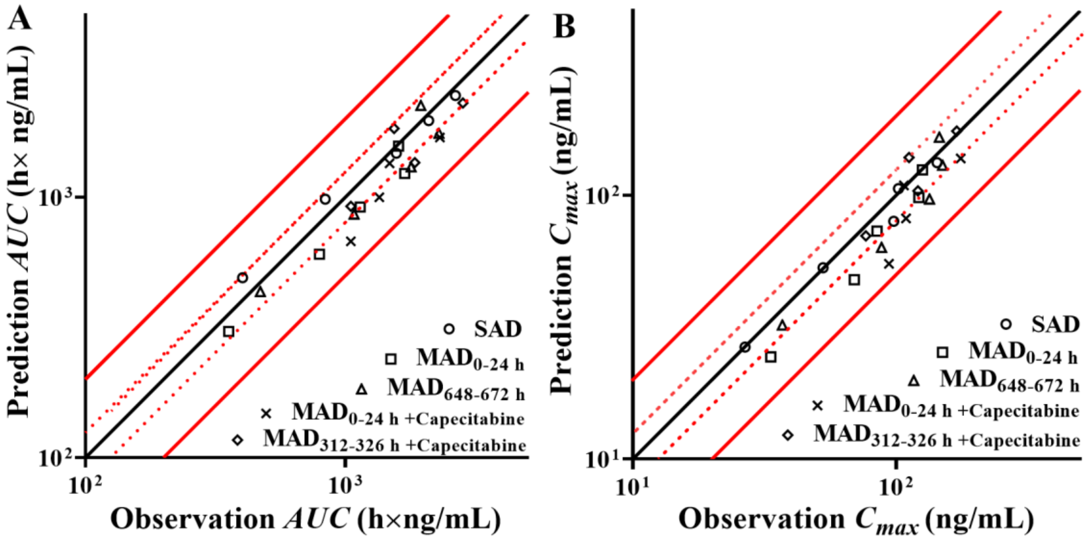

Figure S4 Pharmacokinetic parameters validated results based on single ascending dose (SAD), multiple ascending doses (MAD) and multiple ascending doses of pyrotinib coadministration with capecitabine (MAD+Capecitabine). The red solid lines are predefined 2-fold, the red dashed lines are predefined 1.25-fold boundary

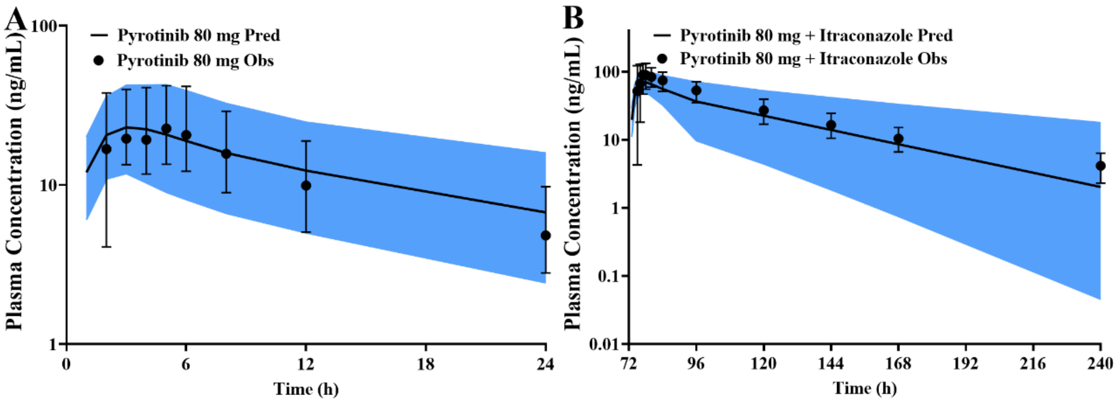

Figure S5 The validated results for pyrotinib exposure in the absence (A) and presence (B) of CYP 3A strong inhibitor itraconazole (The black line is the predicted value, the dark spots are the observed values, and the blue range is 90% CI of the predicted results)

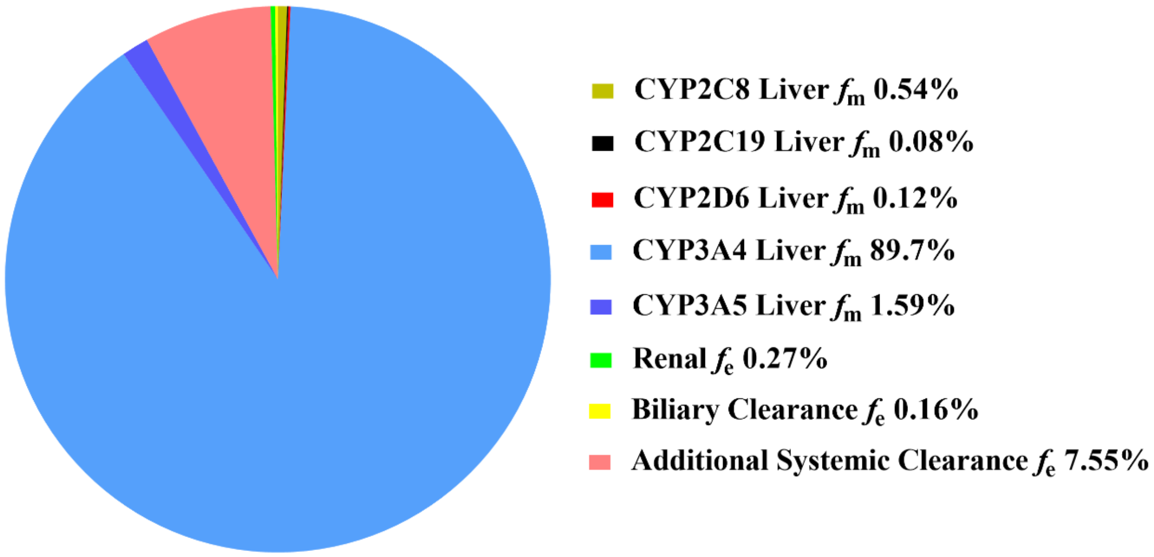

Figure S6 The predicted metabolism/excretion contribution percentage for pyrotinib clearance *in vivo*

Table S1 Model parameters using for pyrotinib PBPK model development

**Parameter**	**Input Value**	**Source**
Physicochemical properties		
Molecular weight (g/mol)	583.08	
log *P*	5.63	Predicted
Compound type	Diprotic base	
*pK*a	12.29, 9.26	Predicted
Blood-to-plasma partition ratio	0.86	
Fraction unbound in plasma	0.053	
Absorption		
Absorption model	ADAM	
Permeability Assay	Caco-2	
Caco-2(10^{-6}cm/s) (pyrotinib)	0.283	Measured
Caco-2(10^{-6}cm/s) (Atenolol)	0.41	Measured
Caco-2(10^{-6}cm/s) (Propranolol)	13.7	Measured
Dissolution Profile		
Time (h) 1/ Dissolution (%) 1	0.00 h /0.00%	Measured
Time (h) 2/ Dissolution (%) 2	0.167 h /43.6%	Measured
Time (h) 3/ Dissolution (%) 3	0.25 h /67.0%	Measured
Time (h) 4/ Dissolution (%) 4	0.33 h /83.4%	Measured
Time (h) 5/ Dissolution (%) 5	0.50 h /97.9%	Measured
Time (h) 6 /Dissolution (%) 6	0.75 h /99.6%	Measured
Distribution		
Distribution model	Full PBPK model	
V_{ss} (L/kg)	9.474	Predicted
Prediction Method	Method 2	
Tissue: Plasma Partition Coefficients (Species defined in Simcyp Animal Simulator, Sim-Rat)		Predicted
Kp scalar	1.80	Fitted
Elimination		
Clearance type	Enzyme kinetics	
Intrinsic clearance of CYP 2C8 (μL/min/pmol)	0.95	Measured
ISEF	0.65	Fitted
Intrinsic clearance of CYP 2C19 (μL/min/pmol)	0.285	Measured
ISEF	0.65	Fitted
Intrinsic clearance of CYP 2D6 (μL/min/pmol)	0.185	Measured
ISEF	0.65	Fitted
Intrinsic clearance of CYP 3A4 (μL/min/pmol)	10.75	Measured
ISEF	0.65	Fitted
Intrinsic clearance of CYP 3A5 (μL/min/pmol)	0.77	Measured
ISEF	0.65	Fitted
Typical renal clearance (L/h)	0.17	Measured
Biliary CL_{int} (Hep) (μL/min/10^6)	0.40	Fitted

Additional Systemic Clearance (L/h)	4.50	Fitted

Table S2 The dosage regimen of pyrotinib combined with itraconazole and the predicted pyrotinib AUCR and $C_{max}R$ (population representative) based on basic pyrotinib PBPK model

Perpetrators	Dosage regimens	Treatment (days)	Pyrotinib dosage regimens (Single dose)	AUCR	$C_{max}R$
(A) Itraconazole	200 mg (D1-D9, QD)	9	80 mg on D4	7.43	1.85
(B) Itraconazole	200 mg (D1, BID; D2-D9, QD)	9	80 mg on D1	9.80	1.82
(C) Itraconazole	200 mg (D1, BID; D2-D9, QD)	9	80 mg on D2	9.34	1.87
(D) Itraconazole	200 mg (D1, BID; D2-D9, QD)	9	80 mg on D3	8.57	1.87
(E) Itraconazole	200 mg (D1-D9, BID)	9	80 mg on D4	8.77	1.94
(F) Itraconazole	200 mg (D1-D14, QD)	14	80 mg on D4	10.85	1.85
(G) Itraconazole	200 mg (D1-D17, QD)	17	80 mg on D4	12.47	1.85
(H) Itraconazole	200 mg (D1, BID; D2-D14, QD)	14	80 mg on D3	11.30	1.87
(I) Itraconazole	200 mg (D1-D7, QD)	7	80 mg on D4	5.45	1.85
(J) Itraconazole	200 mg (D1-D7, QD)	7	160 mg on D4	5.45	1.85
(K) Itraconazole	200 mg (D1-D8, QD)	8	160 mg on D5	5.67	2.03
(L) Itraconazole	200 mg (D1-D9, QD)	9	160 mg on D4	7.43	1.85
(M) Itraconazole	200 mg (D1-D14, QD)	14	160 mg on D4	10.85	1.85
